# Comparative evaluation of antimicrobial efficacy for different root canal irrigants against *Enterococcus faecalis* - An *in vitro* study

**DOI:** 10.6026/973206300222583

**Published:** 2026-04-30

**Authors:** Shah Maruf Hussain, Shipra Jaidka, Deepti Jawa, Alisha Hoda, Shailesh Kumar, Tanushree Basu

**Affiliations:** 1 Department Pediatric and Preventive Dentistry, Divya Jyoti College of Dental Sciences and Research, Modinagar, Uttar Pradesh, India; 2 Department of Dental Surgery, AM Dental Care, Kalkaji, New Delhi, India

**Keywords:** Endodontic irrigants, sodium hypochlorite, BioPure MTAD, silver nanoparticles, *Enterococcus faecalis*

## Abstract

Persistent endodontic infections often involve resistant *Enterococcus faecalis*, making effective root canal irrigation essential for microbial control and treatment success. Therefore, it is of interest to assess antimicrobial efficacy against *E. faecalis* (MTCC 9845) using silver nanoparticles, BioPure MTAD and sodium hypochlorite across 40 Mueller-Hinton agar wells. Sodium hypochlorite demonstrated maximum zone of inhibition, followed closely by BioPure MTAD, while silver nanoparticles showed moderate activity; distilled water served as a negative control with no inhibition. NaOCl remains the gold-standard irrigant due to its superior antimicrobial activity against persistent endodontic pathogens. Thus, BioPure MTAD as a safer, near-equivalent alternative, guiding safer irrigation protocols for resistant infections.

## Background:

Endodontics is the subspecialty of dentistry that focuses on the anatomy, physiology and pathology of the pulp chamber and surrounding tissues. At the core of each tooth lies the dental pulp, a mesenchymal-derived specialized connective tissue that is essential for the tooth's life, nourishment, defense and sensory response [1]. The main cause of pulpal and periapical diseases are believed to be microbial invasion of pulp tissue. Microorganisms and their waste products may enter the pulp space via cavities, injuries and fissures, causing inflammation, necrosis and periapical illness [2]. While resistant facultative organisms like *Enterococcus faecalis* tend to dominate secondary or chronic infections, primary endodontic infections are characterized by polymicrobial and mostly anaerobic infections. A leading cause of post-treatment illness, *E. faecalis* may persist in hostile environments, enter dentinal tubules deeply and resist intracanal medications [3]. Chemical and mechanical preparation, three-dimensional obturation and coronal sealing are the pillars upon which endodontic treatment rests. The intricate structure of root canal systems, particularly in primary teeth, makes mechanical instrumentation inadequate on its own [4]. Thus, irrigation is essential for disinfecting canals, dissolving organic waste and killing microbes. Endodontics is the subspecialty of dentistry that focuses on the anatomy, physiology and pathology of the pulp chamber and surrounding tissues. At the core of each tooth lies the dental pulp, a mesenchymal-derived specialized connective tissue that is essential for the tooth's life, nourishment, defense and sensory response [5]. Microbial invasion of the pulp tissue is considered the primary etiological factor responsible for pulpal and periapical pathologies. Caries, trauma and cracks act as pathways for microorganisms and their by-products to enter the pulp space, leading to inflammation, necrosis and periapical disease [6]. Therefore, it is of interest to evaluate and compare the antimicrobial efficacy of sodium hypochlorite, BioPure MTAD and silver nanoparticles against *Enterococcus faecalis*.

## Materials and Methods:

## Study design:

Research was carried out in the field of Pediatric and Preventive Dentistry using *in vitro* methods.

## Sample size:

Forty wells were prepared on ten Mueller-Hinton agar plates.

## Microorganism:

*Enterococcus faecalis* (MTCC 9845).

## Grouping:

[1] Group 1 - 5.25% Sodium hypochlorite (Positive control)

[2] Group 2 - BioPure MTAD

[3] Group 3 - Silver nanoparticles

[4] Group 4 - Distilled water (Negative control)

After being corrected to 0.5 McFarland standards, the bacterial strain was cultivated in Brain Heart Infusion broth. Following the consistent inoculation of agar plates, wells were made and irrigants were distributed. A Vernier caliper was used to measure the zones of inhibition after incubating the plates at 37°C for 24 hours.

## Sample selection:

## Inclusion criteria:

[1] Pure culture of selected test organism

[2] Plates with uniform lawn culture

[3] Agar plates prepared with correct media

[4] Correctly formed agar wells

[5] Standardized concentration of irrigants

[6] Plates incubated under controlled conditions

## Exclusion criteria:

[1] Contaminated microbial cultures

[2] Uneven lawn formation

[3] Damaged or faulty agar plates

[4] Improper wells

[5] Spillage or leakage of irrigant

[6] Incorrect incubations

[7] Expired or degraded irrigants

[8] Insufficient sample volume

## Results:

The intragroup comparison of mean zones of inhibition across different irrigant groups revealed significant variations in antimicrobial efficacy, with Group 1 (NaOCl, positive control) achieving the highest mean zone of 18.900±0.738 mm (range: 19.000-21.000 mm), followed by Group 2 (BioPure MTAD) at 17.900±0.876mm (range: 16.000-19.000mm), Group 3 (silver nano-particles) at 13.400±1.578 mm (range: 11.000-15.000 mm). Group 4 (distilled water) showed no inhibition at 0.000±0.000 mm (Table 1, Figure 1 - see PDF). A one-way analysis of variance (ANOVA) was performed to compare the mean inhibition zone values among the four study groups: NaOCl (Group 1, positive control), BioPure MTAD (Group 2), Silver nanoparticles (Group 3) and Distilled water (Group 4, control). The analysis revealed a statistically significant difference in mean inhibition zones among the groups (F = 632.281, p = 0.001) (Table 2 - see PDF). Post hoc analysis further revealed a non-significant difference between Group 1 (NaOCl) and Group 2 (Biopure MTAD) (mean difference: 1.000 mm; p = 0.879), indicating comparable antimicrobial efficacy between the two agents. Group 1, however, showed significant superiority over both Group 3 and Group 4, with consistently larger mean zones of inhibition. Similarly, Group 2 demonstrated a significantly greater inhibitory effect than Groups 3 and 4, whereas Group 3 itself was markedly more effective than the control. Accordingly, Groups 1 and 2 emerged as the most potent antimicrobial agents, with similar performance, followed by Group 3, which was moderately effective, while Group 4 exhibited no inhibitory activity (Table 3 - see PDF). The study found that 5.25% sodium hypochlorite exhibited the strongest antimicrobial efficacy against *E. faecalis*, confirming it as the gold-standard endodontic irrigant. BioPure MTAD showed moderate effectiveness, performing better than silver nanoparticles but less than sodium hypochlorite. Silver nanoparticles demonstrated the weakest antibacterial activity, while distilled water showed no inhibitory effect, supporting the validity of the experimental design. The study recommended BioPure MTAD over sodium hypochlorite because it offers effective antibacterial activity against *E. faecalis* while being less cytotoxic and more biocompatible. Unlike sodium hypochlorite, BioPure MTAD does not significantly compromise dentin structure and has a lower risk of tissue irritation, making it a safer and clinically favorable alternative endodontic irrigants.

## Discussion:

Successful endodontic treatment depends on adequate biomechanical preparation, irrigation and three-dimensional obturation of the root canal system. Among these steps, irrigation plays a crucial role because mechanical instrumentation alone cannot eliminate microorganisms from the complex root canal anatomy. This is particularly important in primary teeth, which exhibit complex internal morphology, including accessory canals, furcation communications and horizontal anastomoses, that may harbor microorganisms and limit effective mechanical cleaning [7, 8]. Endodontic infections are polymicrobial, involving both aerobic and anaerobic microorganisms. Among them, *Enterococcus faecalis* is frequently associated with persistent or secondary endodontic infections and is considered a major cause of root canal treatment failure due to its ability to survive harsh environmental conditions and penetrate dentinal tubules. Therefore, effective irrigation protocols are essential for eliminating resistant microorganisms from the root canal system [9]. An ideal root canal irrigant should possess broad antimicrobial activity, the ability to dissolve organic tissue remnants, the capability to remove the smear layer and good biocompatibility with minimal toxicity to surrounding tissues. Over the years, several irrigants have been introduced in endodontics, including sodium hypochlorite, chlorhexidine, EDTA, MTAD and more recently, nanoparticle-based irrigants [10, 11]. In the present study, sodium hypochlorite (NaOCl) demonstrated the highest antimicrobial activity with the largest zone of inhibition against *E. faecalis*. This finding is consistent with previous studies that reported superior antibacterial efficacy of NaOCl compared with other irrigants. The antimicrobial activity of NaOCl is attributed to the formation of hypochlorous acid and free chlorine, which oxidize microbial enzymes and denature proteins, leading to rapid bacterial cell destruction [12]. In addition, NaOCl has the unique ability to dissolve organic tissue remnants, enhancing its effectiveness during root canal disinfection. However, high concentrations of NaOCl may cause cytotoxic effects if extruded beyond the apex, particularly in primary teeth [13]. Similarly, Singh *et al.* (2025) reported that sodium hypochlorite exhibited strong antibacterial activity against *E. faecalis*, and its sequential use with chlorhexidine (with an intermediate rinse of distilled water) resulted in enhanced microbial eradication compared to individual use of irrigants [14]. BioPure MTAD demonstrated antimicrobial efficacy statistically comparable to that of sodium hypochlorite in this study. MTAD is composed of doxycycline, citric acid and a detergent, which together provide antibacterial activity and smear-layer removal [15]. Doxycycline offers sustained antimicrobial activity against *E. faecalis*, while citric acid functions as a chelating agent and the detergent improves penetration into dentinal tubules by reducing surface tension. These combined actions contribute to its effective antimicrobial performance in endodontic disinfection [16]. Silver nanoparticles (AgNPs) also demonstrated antimicrobial activity, but produced the smallest zone of inhibition among the tested agents. The antibacterial action of AgNPs is mainly due to their ability to attach to bacterial cell membranes, release silver ions and generate reactive oxygen species that damage proteins and DNA [17]. However, their antimicrobial effect is influenced by factors such as particle size, concentration and contact time. Limited diffusion of nanoparticles in agar media may also contribute to the smaller inhibition zones observed [18]. Distilled water used as a negative control did not produce any inhibition zone, confirming the validity of the experimental design. Overall, the results indicate that sodium hypochlorite exhibited the highest antimicrobial activity, followed by BioPure MTAD and silver nanoparticles. Although NaOCl remains the gold-standard irrigant, MTAD provides comparable antimicrobial efficacy and silver nanoparticles show promising potential but require further investigation for clinical application in endodontic irrigation systems [19].

## Conclusion:

Sodium hypochlorite was found to be the most effective agent against *Enterococcus faecalis* among all tested irrigants in this *in vitro* investigation. BioPure MTAD emerged as an excellent alternative, offering comparable antibacterial efficacy to conventional regimens with superior biocompatibility. Silver nanoparticles showed the lowest antibacterial effectiveness, indicating limited utility as a primary endodontic irrigant in this model.
